# Distortion correction using topup algorithm by single k-space (TASK) for echo planar imaging

**DOI:** 10.1038/s41598-023-46163-3

**Published:** 2023-10-31

**Authors:** Seon-Ha Hwang, Hyun-Soo Lee, Seung Hong Choi, Sung-Hong Park

**Affiliations:** 1 MRI Laboratory, Department of Bio and Brain Engineering, Korea Advanced Institute of Science and Technology, Daejeon, South Korea; 2 Siemens Healthineers, Seoul, South Korea; 3 Department of Radiology, Seoul National University College of Medicine, Seoul, South Korea; 4 Department of Bio and Brain Engineering, Korea Advanced Institute of Science and Technology, Rm 1002, CMS (E16) Building, 291 Daehak-ro, Yuseong-gu, Daejeon, 34141 South Korea

**Keywords:** Magnetic resonance imaging, Functional magnetic resonance imaging

## Abstract

Distortion of echo planar imaging (EPI) can be corrected using B_0_ field maps, which can be estimated with the topup algorithm that requires two EPI images with opposite distortions. In this study, we propose a new algorithm, termed topup algorithm by single K-space (TASK), to generate two input images from a single k-space for the topup algorithm to correct EPI distortions. The centric EPI contains the opposite phase-encoding polarities in one k-space, which can be divided into two halves with opposite distortions. Therefore, two inputs could be extracted by dividing the k-space into halves and processing them using the proposed procedure including an iterative procedure of automatic brain masking and uniformity correction. The efficiency of TASK was evaluated using 3D EPI. Quantitative evaluations showed that TASK corrected EPI distortion at a similar level to the traditional methods. The estimated field maps from the conventional topup and TASK showed a high correlation ($r=0.80\pm 0.05$). An ablation study showed the validity of every suggested step. Furthermore, it was confirmed that TASK was effective for distortion correction of two-shot centric EPI as well, demonstrating its wider applicability. In conclusion, TASK can correct EPI distortions by its own single k-space information with no additional scan.

## Introduction

Echo planar imaging (EPI)^1^ is a representative fast magnetic resonance imaging (MRI) technique having k-space trajectory (the pattern that the MRI scanner follows when collecting data in k-space) with alternating readout directions filled by a single or several radio frequency (RF) excitations. The main magnetic field (B_0_) inhomogeneity causes distortions in the EPI images due to low bandwidth along the phase-encoding direction. The EPI distortions can be corrected by a B_0_ field map, which contains information about the amount of pixel-wise distortion. There are two representative ways to obtain a B_0_ field map: double-echo gradient echo (GRE) method^2^ and topup algorithm^3,4^. The former^2^ calculates the field map by dividing the phase difference map between the two GRE images by the echo time (TE) difference. It has been considered the gold standard for the B_0_ field mapping, but requires an additional field map scan. The topup algorithm^3,4^ has been suggested to save the additional scan time. This algorithm works based on the fact that the distortion direction of EPI images is determined by the polarity of the phase-encoding blips; The EPI images with opposite blip polarities exhibit distortion in opposite directions. That is, the pixel-wise distortion information can be estimated by comparing two input images with opposite distortions, eventually leading to the B_0_ field map. Since this method obtains one corrected image using two images with opposite distortions, the temporal resolution is reduced by a factor of two or higher. Therefore, in general, a single image with opposite distortion is acquired before or after the main EPI scan to maintain temporal resolution. This approach still requires an additional scan, even if the additional scan time is much shorter. However, since the B_0_ field map may change unexpectedly during the EPI scan due to motion or field drifting, these conventional methods that require the additional scan cannot address the dynamic B_0_ field map changes.

There have been several strategies to enable dynamic field mapping and distortion correction for EPI. Wallace et al*.* proposed navigators for free induction decay to enable dynamic distortion correction while minimizing the temporal resolution loss^5^. It had verified correction performance, but navigators must be inserted in imaging sequence. Liao et al*.* showed dynamic field mapping by its own single k-space scanned by interleaved blip-up/down phase encoding^6^. Although this method permitted dynamic mapping without additional navigator, applications are limited to multi-shot EPI, which is not suitable for the functional MRI and magnetization-prepared imaging requiring high temporal resolution. Additionally, there have been deep learning methods for dynamic corrections, where the networks are trained to generate distortion corrected images or field maps^7–11^. However, deep learning networks typically demand extensive training data to ensure reliability and may encounter challenges with generalization when applied to varying scan parameters, organs, and scanners.

**Figure 1:**
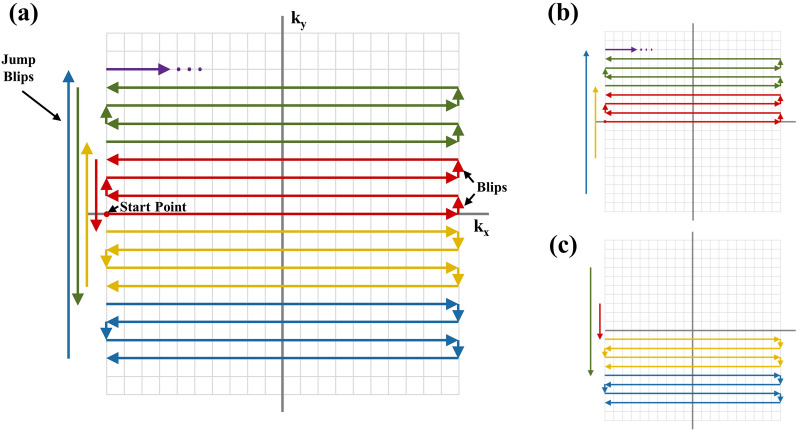
k-space trajectory of 3D 1sh-CenEPI. (**a**) The k-space trajectory. The k-space was sequentially imaged along red, yellow, green, and blue lines. (**b,c**) Dividing the k-space into two parts according to the blip polarity. The upper k-space (**b**) and the lower k-space (**c**) consist of the positive blip polarity and the negative blip polarity, respectively.

If the topup algorithm can be implemented with a single image, it would enable us to resolve the disadvantage of necessity of an additional scan or lower temporal resolution, leading to another dynamic field mapping strategy. In this study, we propose to implement the topup algorithm using a single image from the centric EPI^12,13^. The centric EPI provides higher signal-to-noise ratio (SNR) because the centric part of k-space is scanned first, but it has been considered possible only with multi-shot imaging due to the requirement of small EPI blip gradients connecting the phase-encoding lines^13^. However, recently, unique centric phase-encoding trajectories have been proposed for EPI^12^ and balanced steady state free precession^14^. The former enables us to get single-shot pseudo-centric EPI (1sh-CenEPI), where the k-space trajectory is designed to achieve centric-ordered EPI with a single RF excitation (Fig. 1). In 1sh-CenEPI, the phase encoding order was revised in a pseudo-centric manner by alternately imaging the upper/lower k-space halves in the opposite blip polarities and applying jump blips in every certain number of phase-encoding lines (N). Since the jump blips have a little longer duration and higher magnitude than ordinary ones, additional eddy currents and varying readout window can contribute to the phase accumulations, resulting in additional distortion. The previous study compensated for the phase accumulations by the whole-echo correction method and proposed an optimal N value (N = 4) that minimizes the eddy current effect while preserving the magnetization-preparation contrast.

For both multi-shot and single-shot centric EPIs to achieve centric order, the k-space trajectory has opposite blip polarities in two k-space halves, leading to opposite distortions coexisting in a single k-space. For the centric EPI images, the distortion should be corrected separately for each k-space half in the opposite direction using additional field map scan, and then the two corrected k-space halves should be merged^12^. In another perspective, two images with opposite distortions can be derived by dividing a single k-space of centric EPI into two halves, potentially being used as two input images of the topup algorithm for distortion correction with no additional scan.

This paper addresses the process for deriving two input images for the topup algorithm from a single EPI k-space and applying them to existing topup for distortion correction. This study will call this process as topup algorithm by single k-space (TASK). Multiple processing steps are proposed to obtain the two optimal input images from a single k-space of 1sh-CenEPI, including automatic brain mask construction and uniformity correction. In addition, their roles were investigated by an ablation study. Then, we demonstrated that by applying the two derived input images from a single k-space of 1sh-CenEPI to the FSL’s topup algorithm^15^, the B_0_ field map can be estimated and applied for the distortion correction. The 1sh-CenEPI was applied as a readout sequence of pseudo-continuous arterial spin labeling (pCASL)^16^ to demonstrate the efficiency and advantage of the proposed TASK for magnetization-prepared imaging. The performance of the TASK was verified by comparison with distortion-free brain boundary and corrected images by the conventional topup. Also, quantitative comparisons were conducted with Dice coefficient, Hausdorff distance, cerebral blood flow (CBF) values, SNR and correlation. Moreover, the utility of TASK was also validated for two-shot centric EPI^13^, to demonstrate its potential broader applications.

## Methods

### Pulse sequence

**Figure 2:**
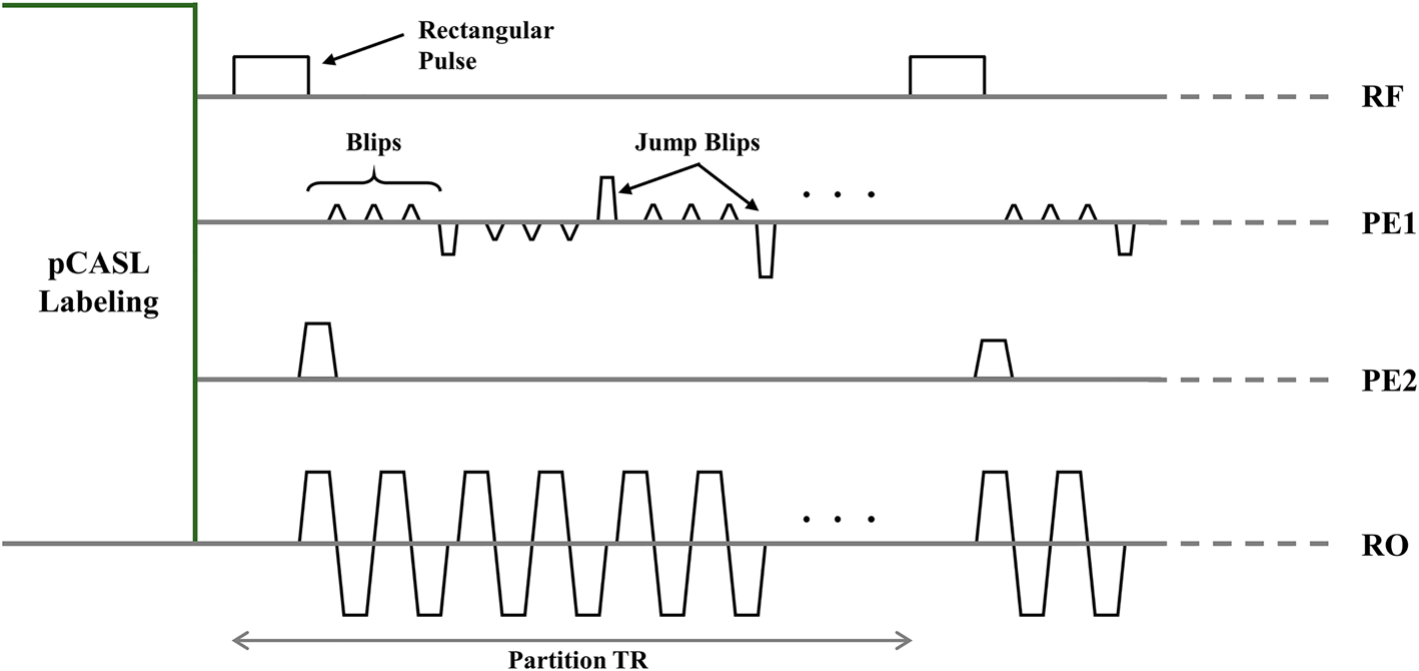
Sequence diagram of 3D 1sh-CenEPI-pCASL. After the pCASL labeling scheme, 3D 1sh-CenEPI was conducted. Water-excitation rectangular RF pulses were applied in every kx–ky plane. Jump blips were applied in every four phase-encoding lines.

The MRI pulse sequence starts with the (I) the pCASL magnetization-preparation part followed by (II) the 3D 1sh-CenEPI readout part (Fig. 2). Along the k_z_ direction, the 3D 1sh-CenEPI was acquired in an ascending order with as many RF excitations as the number of the corresponding k_z_ encoding steps. Each k_z_ plane was imaged in single-shot following 1sh-CenEPI trajectory^12^. The 1sh-CenEPI trajectory filled the single k_z_ plane from the center to the edge part of the k-space by a single RF excitation with jump blips (Fig. 1). Following the recommendation of the previous study^12^, a jump blip was inserted every four phase-encoding lines to balance SNR, contrast of the magnetization preparation, and the number of big jump blips that can cause eddy current artifacts.

### Data acquisition

The experiments, approved by Institutional Review Board of KAIST (IRB #KH2022-138), were performed on nine subjects using 3T MRI (Skyra, Siemens, Erlangen, Germany) with a 64-ch head coil. Written informed consent was obtained from all subjects. All methods were performed in accordance with the relevant guidelines and regulations. The parameters for the pCASL part were as follows: RF duration/spacing = 0.5/1 ms; label offset = 90 mm; effective post labeling delay = 1.5 s; flip angle = 25$^\circ$; labeling duration = 1.8 s; maximum/mean slice selective gradients = 6/1 mT/m; Three inversion recovery pulses were applied to suppress background. The labeling plane was located at the base of cerebellum. Additionally, the EPI images with the same repetition time (TR) but without the pCASL scheme (i.e., magnetization transfer (MT)-free M_0_ images) were acquired 15 times and then averaged for CBF quantification^16^.

The parameters for the 3D 1sh-CenEPI readout part were as follows: Matrix size = 64 × 64 × 40; field of view = 230 × 230 × 200 mm^3^; partition TR = 39 ms (minimum); TE = 5.6 ms (minimum); flip angle = 12$^\circ$ (Ernst angle for gray matter, T_1,GM_ = 1820 ms); receiver bandwidth = 1910 Hz/pix; phase-encoding direction = A-P; k_z_ ordering = linear; total TR = 4.25 s, and total scan time < 5 min (including 34 pairs of label and control scans followed by 15 measurements of MT-free M_0_ images). Additionally, partial Fourier was applied for both phase and partition encoding directions to avoid the large jump blips and decrease the data readout time^12^. Furthermore, non-selective water-excitation rectangular RF pulses with 4919 $\mathrm{\mu s}$ duration were used to suppress fat with no additional pulse (Fig. 2)^17^.

For the comparison, conventional 3D linear EPI, where the k-space was filled in the linear ascending order along the phase-encoding direction, was also scanned with the pCASL scheme. The scan parameters of the linear EPI were the same as those of 3D 1sh-CenEPI except 15.3 ms TE and 30 ms partition TR. Additionally, for the comparison with the conventional topup, the linear EPI with opposite blips was imaged separately in four subjects.

To test the generalizability of the TASK in terms of imaging parameters, an additional gradient-echo 2D 1sh-CenEPI images were acquired in one subject. The scan parameters were as follows: Matrix size = 96 × 96; field of view = 192 × 192 mm^2^; slice thickness = 5 mm; the number of slices = 10; flip angle = 35$^\circ$; receiver bandwidth = 1580 Hz/pixel; TR = 300 ms; TE = 1.4 ms; RF pulse = Asymmetric (minimum phase). The short TE was possible by combination of an asymmetric RF pulse and the pseudo-centric ordering.

To get the ground truth brain boundaries and B_0_ field maps, 2D double-echo GRE images with TEs of 4.92 ms and 7.38 ms were obtained. The TEs were determined by considering phase conditions of fat and water as recommended by the vendor. TR was 12 ms, flip angle = 25$^\circ$, and the other parameters were the same as those of the EPI.

Instead of the conventional 3-echo correction method^18^ where the center three phase-encoding lines are acquired as the reference scan, the whole-echo correction^12^ was conducted for 1sh-CenEPI to eliminate the N/2 ghost. In the whole-echo correction, the reference scan was conducted for all phase encoding lines, having the same echo spacings as the main EPI scan. This reference scan was conducted only once before the main scan not to compromise the temporal resolution. Therefore, it could compensate for not only N/2 ghost, but also the inconsistent echo spacings between two adjacent phase-encoding lines caused by jump blips. That is, the whole-echo correction compensated for the additional distortion due to inconsistent echo spacings, so that the 1sh-CenEPI images could have the same amount of distortion as the linear EPI, as demonstrated in the previous study^12^.

### Field map estimation

**Figure 3:**
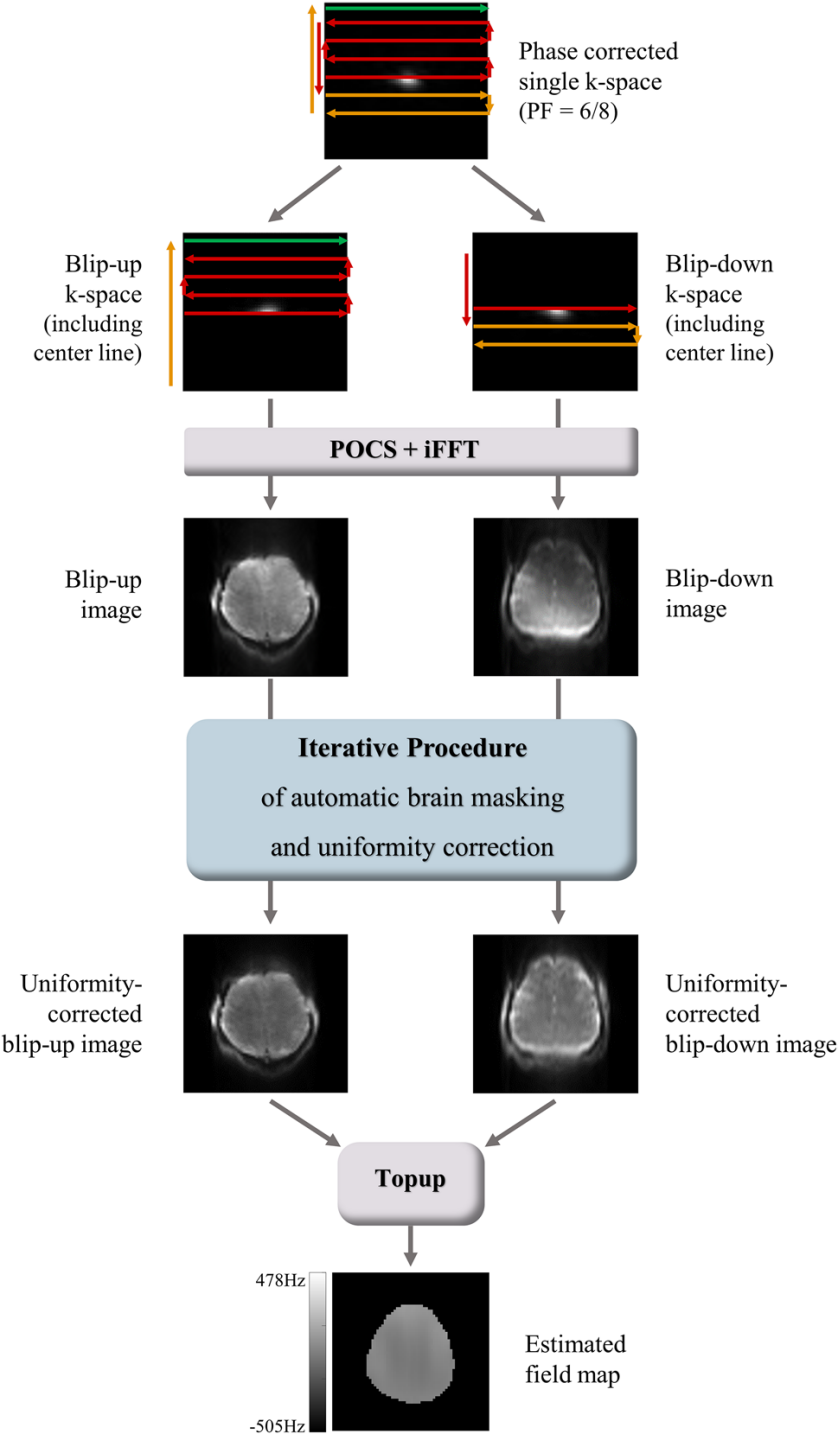
Overall process to estimate B_0_ field map. A single phase-corrected k-space was divided into two halves and reconstructed to images by POCS. To compensate for the signal suppression by insufficient k-space information, an iterative procedure of automatic brain masking and uniformity correction was applied. Finally, the topup method with the two final input images with opposite distortions could estimate the B_0_ field map.

Simply dividing a single k-space of 1sh-CenEPI into two halves (Fig. 1b,c) resulted in two images with insufficient quality for the topup algorithm due to incompleteness of partial k-spaces. In this study, a new process for constructing two input images for the topup algorithm from a single k-space is suggested, which is illustrated in Fig. 3. The B_0_ field map was estimated with the first-measured phase-corrected 3D k-space by 1sh-CenEPI^12^ without the pCASL scheme (i.e., the first-measured MT-free M_0_ images among the 15 repetitions) and then it was used for distortion correction of all the k-spaces of the 1sh-CenEPI without and with the pCASL scheme (i.e., all the 15 MT-free M_0_ images and the perfusion-weighted images). For the field map estimation, the single k-space of the first-measured 1sh-CenEPI was divided into two k-space halves (blip-up and blip-down) with zero filling to match the initial image size. In this step, the center line of k-space (the first scanned line not affected by any gradient blip) was included in both to balance the signal intensity between the two input images. In the next step, zero-filled opposite half (blip-up) and quarter (blip-down, due to partial Fourier) of two respective separated k-spaces were updated by projection onto convex sets (POCS)^19,20^. It compensated for phase inconsistency of the partial k-spaces, mitigating aliasing-like artifacts. Subsequently, by inverse Fourier transforming two extracted k-spaces, two preliminary input images were generated.

However, there was signal intensity mismatch between two input images due to signal suppression by incompleteness of partial Fourier^21^, resulting in poor performance of the topup algorithm. To compensate for the signal suppression, an iterative procedure of automatic brain masking and uniformity correction was proposed and applied to both blip-up and down images.

**Figure 4:**
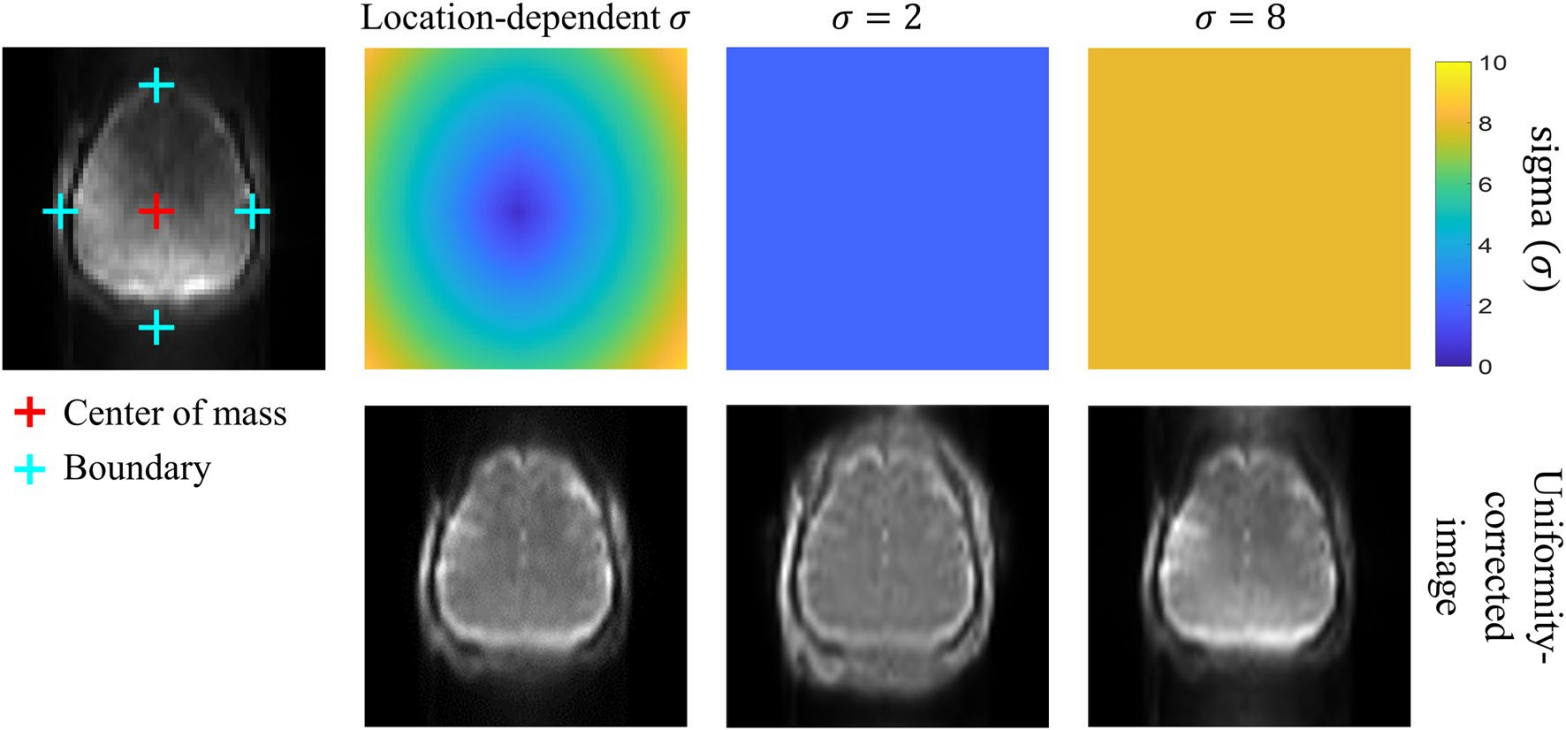
Uniformity-corrected images depending on the sigma values. For the location-dependent uniformity correction, the center of mass of the brain (red cross) and the boundary points (cyan cross) (where the vertical and horizontal lines passing the center of mass meet the brain boundary) were found automatically. Then, the sigma values were assigned following the location information. For estimation of the final B_0_ field map, the location-dependent correction achieved superiority in both the brain boundaries with non-artificial signals and the inner brain regions with homogeneous signals. A single high sigma value ($\upsigma =8$) was used for accurate brain boundary of automatic brain masking in the iterative procedure explain in Fig. 5.

**Figure 5:**
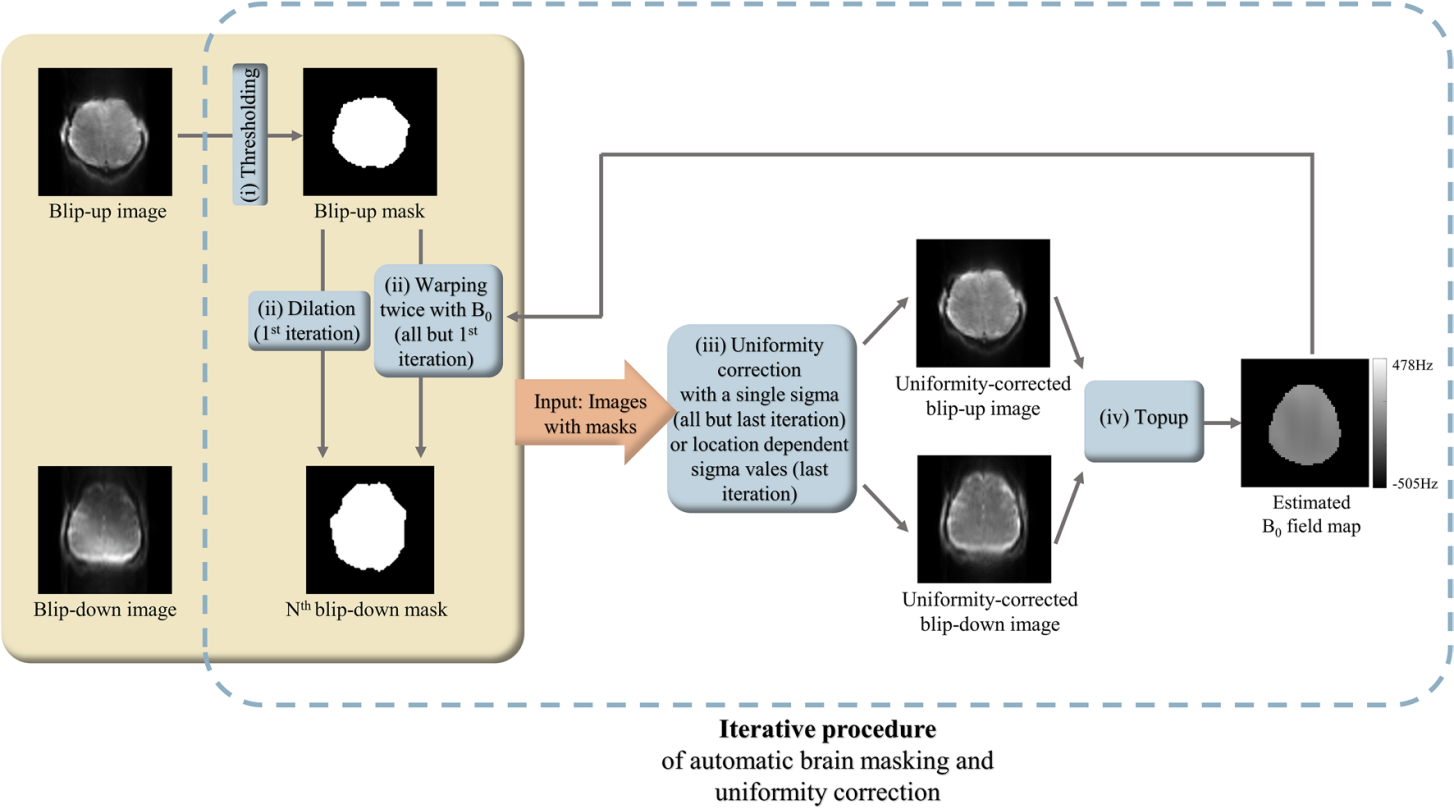
Schematic diagram of the iterative procedure of automatic brain masking and uniformity correction. It was designed to derive more accurate brain mask for the blip-down image, eventually leading to estimation of more accurate final B_0_ field map.

The uniformity correction^22^ was achieved by dividing the original image by its lowpass-filtered image. The lowpass-filtered image was calculated by applying Gaussian smoothing to a target image where the object part (brain in this study) was maintained as the original image and the background part was replaced with the maximum value to suppress the background noise. The object and the background parts were determined by automatic brain masking explained in the next section. Higher sigma values for the smoothing (higher smoothing) were desirable at the brain boundary to prevent the abrupt signal changes in the lowpass-filtered image, whereas lower sigma values (less smoothing) were desirable in the inner brain regions for better signal homogeneity (Fig. 4). The sigma value for the Gaussian smoothing was determined to be either (i) a constant high value ($\upsigma =8$) for an accurate brain boundary in the automatic brain masking to better estimate the preliminary B_0_ field map or (ii) a location-dependent manner (location-dependent uniformity correction), where the sigma values were determined empirically and automatically to be 0.5 at the center of mass and 8 at the brain boundary and to be linearly increasing in between, for the accurate final B_0_ field map estimation (Figs. 4, 5). Eventually, two input images with opposite distortions were constructed by dividing the original image by the lowpass-filtered image with the specified sigma value(s) to mitigate the signal mismatch (Fig. 3). It should be noted that the uniformity correction was applied only for the B_0_ field map estimation and thus there was no artificial effect on the final distortion-corrected EPI images. Finally, the B_0_ field map was estimated by subjecting the extracted two input images to the conventional topup procedure by the *topup* function in FSL^15^. The code was as follows: topup –imain = abs_topup.nii –datain = acq.txt –config = b02b0.cnf –estmov = 0 –out = topup_result, where abs_topup.nii was two input images and acq.txt was the imaging information including the phase encoding direction.

### Iterative procedure of automatic brain masking and uniformity correction

In the uniformity correction step, automatic brain masks for the blip-up and down images were necessary to distinguish the object from the background. Otherwise, uniformity correction process would abnormally increase the background noise. Traditional masking methods such as intensity threshold^23^ and brain extraction tool^15,24^ were not suitable for the blip-down image due to the signal suppression by incompleteness of partial k-space or EPI distortion. Therefore, the iterative procedure of automatic brain masking and uniformity correction was proposed for the blip-down image (Fig. 5): (i) Due to the better image quality, a brain mask for the blip-up image was first calculated by Otsu’s intensity thresholding^25^. (ii) The brain mask of the blip-down image was estimated either by dilation^26^ of the blip-up mask with a triangular structural element (first iteration, no B_0_ field information) or by warping the blip-up mask twice using the estimated B_0_ field map because of opposite distortions between the two (all but the first iteration). (iii) The uniformity correction with either the constant high sigma value ($\upsigma =8)$ (all but the last iteration) or the location-dependent sigma values (last iteration) was applied to the blip-up and blip-down images using their preliminary corresponding brain masks. (iv) The (preliminary or final) B_0_ field map was estimated by the topup algorithm^3^ with the uniformity-corrected blip-up and blip-down input images. To generate the field map with more accurate blip-down mask, the steps from (ii) to (iv) were repeated total three times, which was empirically sufficient in this study. The final B_0_ field map was used for the distortion correction of the 1sh-CenEPI images as explained in the next section. Again, the proposed iterative procedure of automatic brain masking and uniformity correction was applied only for the B_0_ field map estimation and thus would not cause any artificial effect on the final distortion-corrected images.

### Distortion correction

For the distortion correction with the estimated field map, the field map should be applied in opposite directions for each half of the k-space, because opposite distortions coexist in a single k-space of 1sh-CenEPI. Following the previous research^12^, the k-space of 1sh-CenEPI was divided into two halves as blip-up and blip-down k-spaces (which had opposite distortions). Unlike in the process of B_0_ field map estimation, the center k-space line was included only in the blip-up k-space. The other halves of the two k-spaces were simply filled with zeros. Then, the distortion was corrected for the complex images of blip-up and blip-down separately in the opposite direction using the final B_0_ field map mentioned above. The final distortion-corrected image was produced by complex averaging of the corrected blip-up and down images and by least square restoration^27^. This process was conducted by the *applytopup* function in FSL^15^. The code was as follows: applytopup –imain = up.nii,down.nii –datain = acq.txt –inindex = 1,2 –topup = topup_result –out = real_unwarped.nii.gz –method = lsr, where up.nii and down.nii were the blip up and down images, respectively.

For the comparison reference, the B_0_ field maps were additionally measured using the standard double-echo GRE method^2^ and the conventional topup method^3^. For the double-echo GRE method, the B_0_ field map was calculated following the convention (dividing the phase difference map by the TE difference). The FSL^15^ code for applying the field map estimated by this conventional method was as follows: fugue -i epi_lin –dwell = dwelltime –loadfmap = fieldmap -u epi_unwarp, where epi_lin was the linear EPI images and fieldmap was the estimated field map by the double-echo GRE method. For the conventional topup method, the B_0_ field map was estimated by the *topup* function in FSL using the two EPI images with linear phase-encoding order of opposite polarities^15^.

### Evaluation of the TASK

For the analysis of the results, the distortion-free brain boundary was obtained by applying intensity thresholding and skull stripping on the GRE images. Lastly, to verify the efficiency on perfusion-weighted imaging, CBF was measured by the pCASL as follows:

$$\mathrm{CBF}= \frac{\Delta M}{{M}_{0}}\cdot \frac{6000\cdot \lambda \cdot {e}^{\frac{PLD}{{T}_{1,blood}}}}{2\cdot \alpha \cdot {T}_{1,blood}\cdot (1-{e}^{-\frac{\tau }{{T}_{1,blood}}})} [\text{ml/100} \, \text{g/min}],$$

where ${T}_{1,blood}$ is the longitudinal relaxation time of blood (${T}_{1,blood}=1.65\text{ s}$), $\alpha$ is the labeling efficiency ($\alpha =0.68)$, $\lambda$ is the brain-blood partition coefficient ($\lambda =0.9\text{ ml/g}$), $PLD$ is the post labeling delay ($PLD=1.5\text{ s}$), $\tau$ is the labeling duration ($\tau =1.8\text{ s}$), $\Delta M$ is the average signal difference between label and control images, and ${M}_{0}$ is the average signal of MT-free EPI images, which are acquired without the pCASL preparation^16^.

For the quantitative comparison, perfusion SNR^28^, perfusion temporal SNR (tSNR)^29^, Dice coefficient^30^, Hausdorff distance^31^ and correlation^32^ were measured and statistical significance was tested with a two-tailed t-test. The perfusion SNR was calculated as spatial SNR of perfusion-weighted images, (i.e., gray matter perfusion signals/background standard deviation). The perfusion tSNR was calculated by dividing the average gray matter temporal signal from perfusion-weighted images by the temporal standard deviation. A gray matter mask was manually generated and used to calculate the average value of the gray matter area. The Dice coefficient and Hausdorff distance were calculated between brain masks from the distortion-corrected and GRE (i.e., ground truth) images, indicating how similar the two brain masks were. In other words, it showed distortion correction ability of the TASK by comparing the brain boundary with the ground truth. The brain masks for Dice coefficient and Hausdorff distance were first drawn with intensity thresholds^23^, followed by skull-stripping. In addition, the Pearson’s product moment correlation between estimated B_0_ field maps by the TASK and the conventional topup method was calculated within the brain regions of the four subjects. It was calculated at the five center slices for consistent comparisons across all subjects regardless of brain size. The correlation with the double-echo GRE method was not compared because it generates B_0_ field maps using an algorithm different from the conventional topup and TASK methods, and using a dataset different from that of the TASK.

Furthermore, the TASK was applied to the 2D 1sh-CenEPI image with higher in-plane resolution (96 × 96 matrix size) to check its generalizability to the various imaging parameters. Lastly, to evaluate contributions of each step of the proposed field map estimation, a qualitative visual evaluation by an ablation study was performed. The proposed steps were added one by one and each resulting image was compared.

## Result

**Figure 6:**
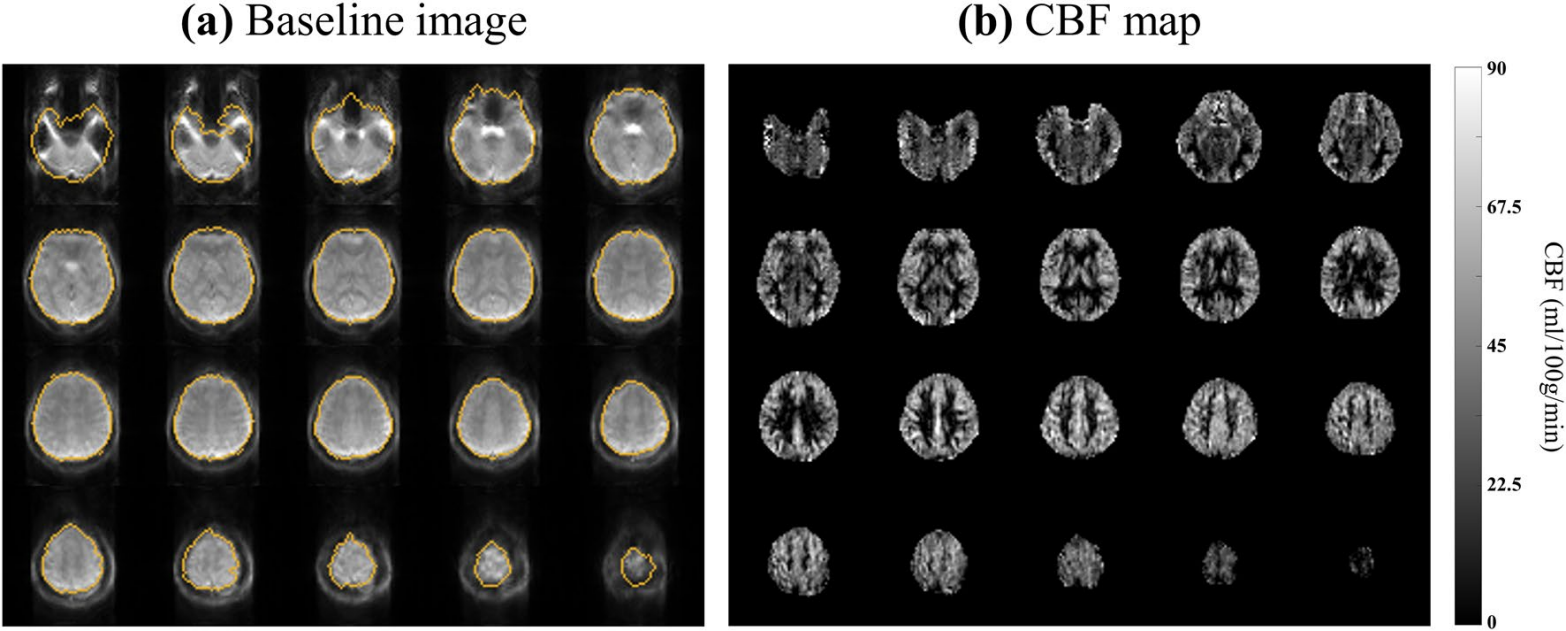
Whole-brain (**a**) baseline image and (**b**) perfusion map from a representative subject (Subject #1). (**a**) The orange lines represent the ground truth brain boundary from GRE images.

**Figure 7:**
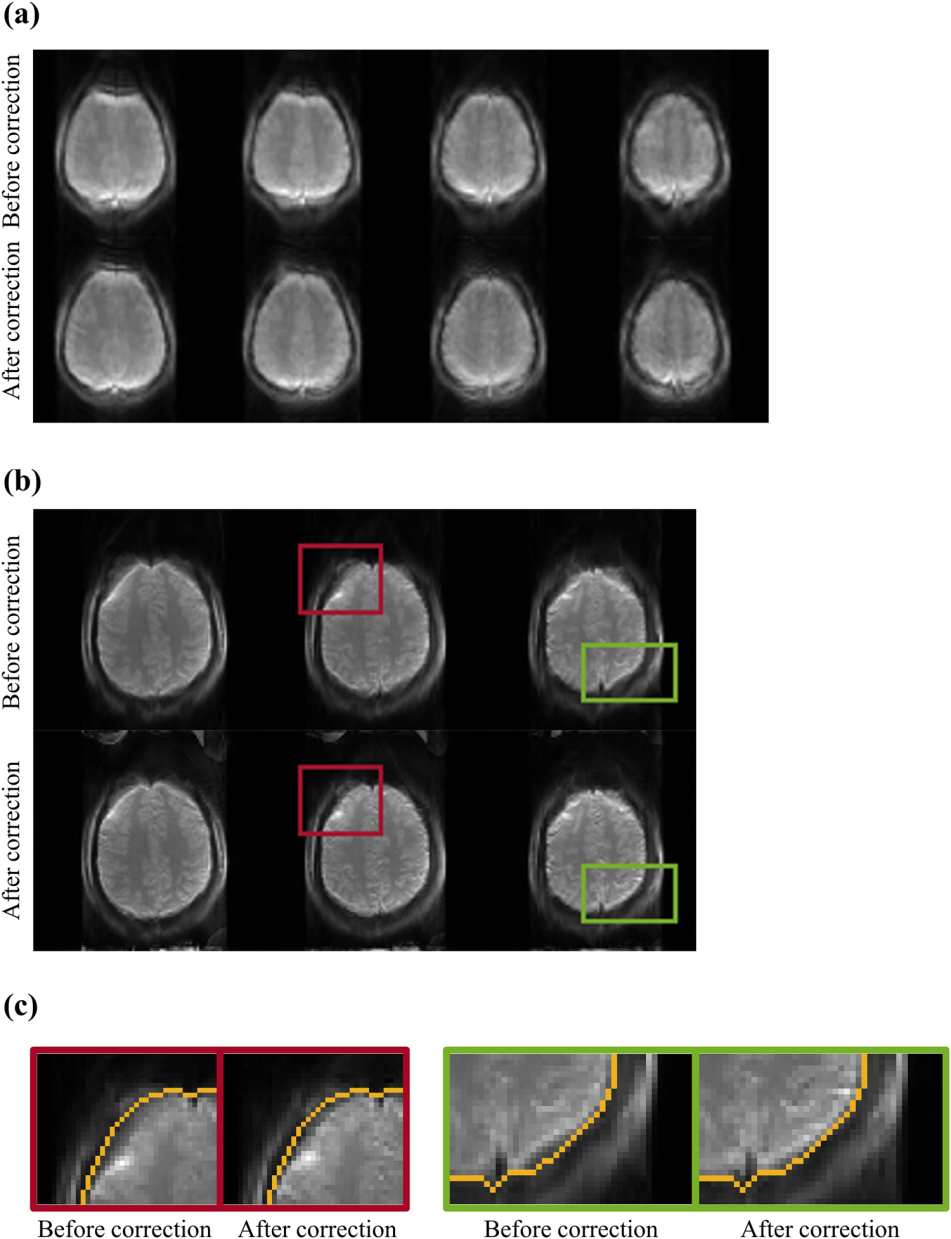
Comparison between before and after the distortion correction. The comparison was conducted for both (**a**) 3D 1sh-CenEPI in Subject #2 and (**b**) 2D 1sh-Cen EPI with matrix size of 96 × 96. (**c**) Magnified images of 2D 1sh-Cen EPI images are shown with the ground truth brain boundary from GRE images (orange line).

The representative distortion-corrected images and CBF maps are shown in Fig. 6. Most of the boundaries were recovered well except the regions of severe distortions. CBF maps showed that the fine brain structures were preserved well with no spoil by the correction process, indicating that the TASK is effective in magnetization-prepared imaging. Comparison between the images before and after the TASK (Fig. 7a) showed that the coexisting opposite distortions were compensated and merged into the corrected boundary by the TASK. Figure 7b,c shows the feasibility of the TASK for 2D 1sh-CenEPI with higher resolution, which corrected the distorted brain boundary close to the GRE-based ground truth (orange line).

**Figure 8:**
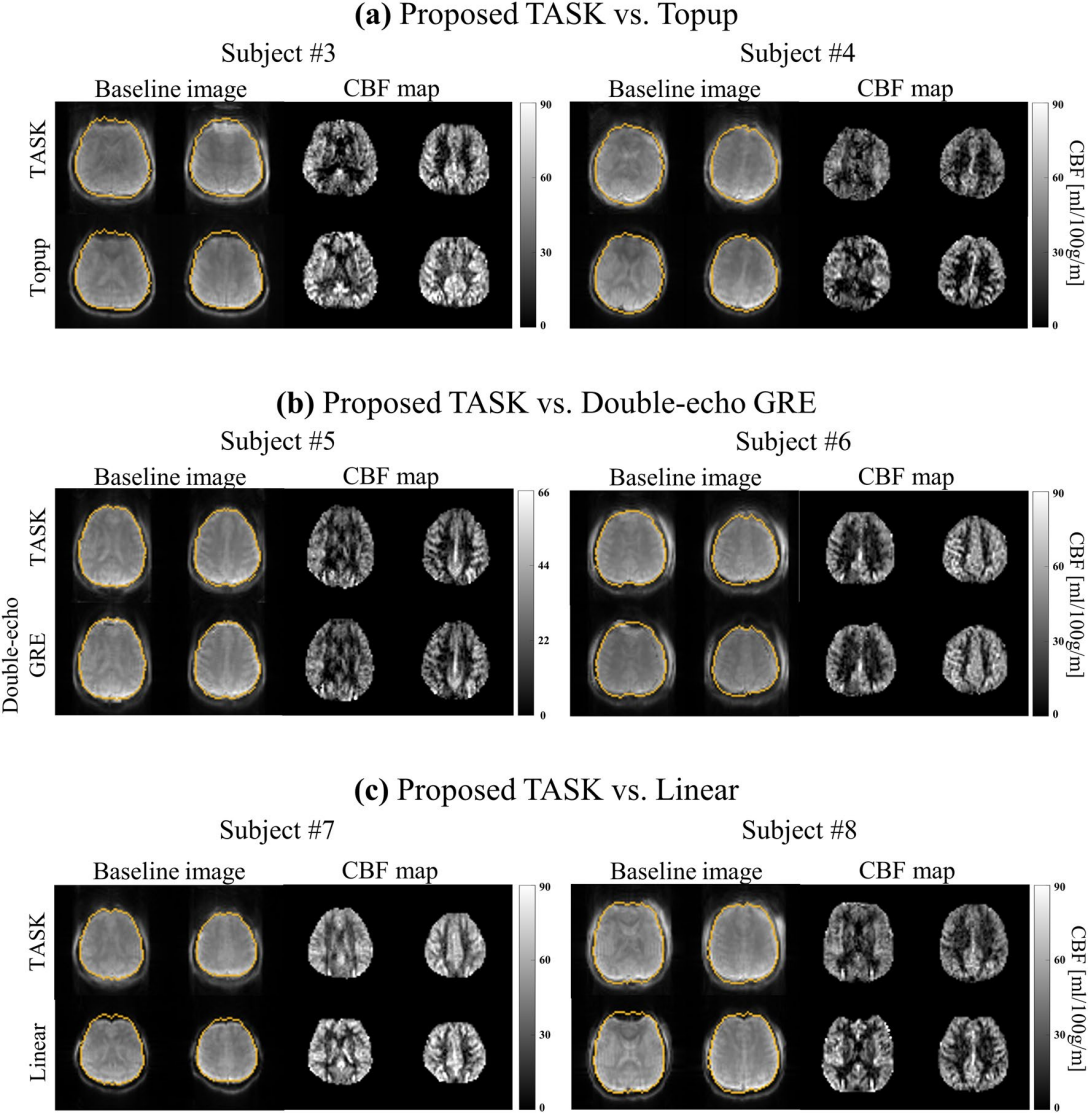
Comparisons of distortion-corrected images. Two representative slices (15 mm apart center slices) of each subject are presented. Images corrected by the TASK were compared with those corrected by (**a**) conventional topup and (**b**) double-echo GRE method and (**c**) those acquired with linear phase-encoding order without any distortion correction.

The performance of the TASK was verified on six subjects as shown in Fig. 8. Two representative center slices (15 mm apart) on each subject are presented. The ground truth brain boundary was extracted from the distortion-free GRE image (orange line). The ground truth CBF map could be considered as the one from 1sh-CenEPI corrected by the double-echo GRE method. Across all the tested subjects, the brain boundaries were well recovered while preserving the inner detailed structure. Comparisons with the conventional topup (Fig. 8a) and the double-echo GRE method (Fig. 8b) show the efficiency of the TASK. Despite its advantage of not requiring additional scans, the TASK showed similar performance to the conventional methods. Furthermore, comparison with the linear EPI images showed superiority of the TASK in distortion correction under the condition of no additional scan (Fig. 8c).

**Figure 9:**
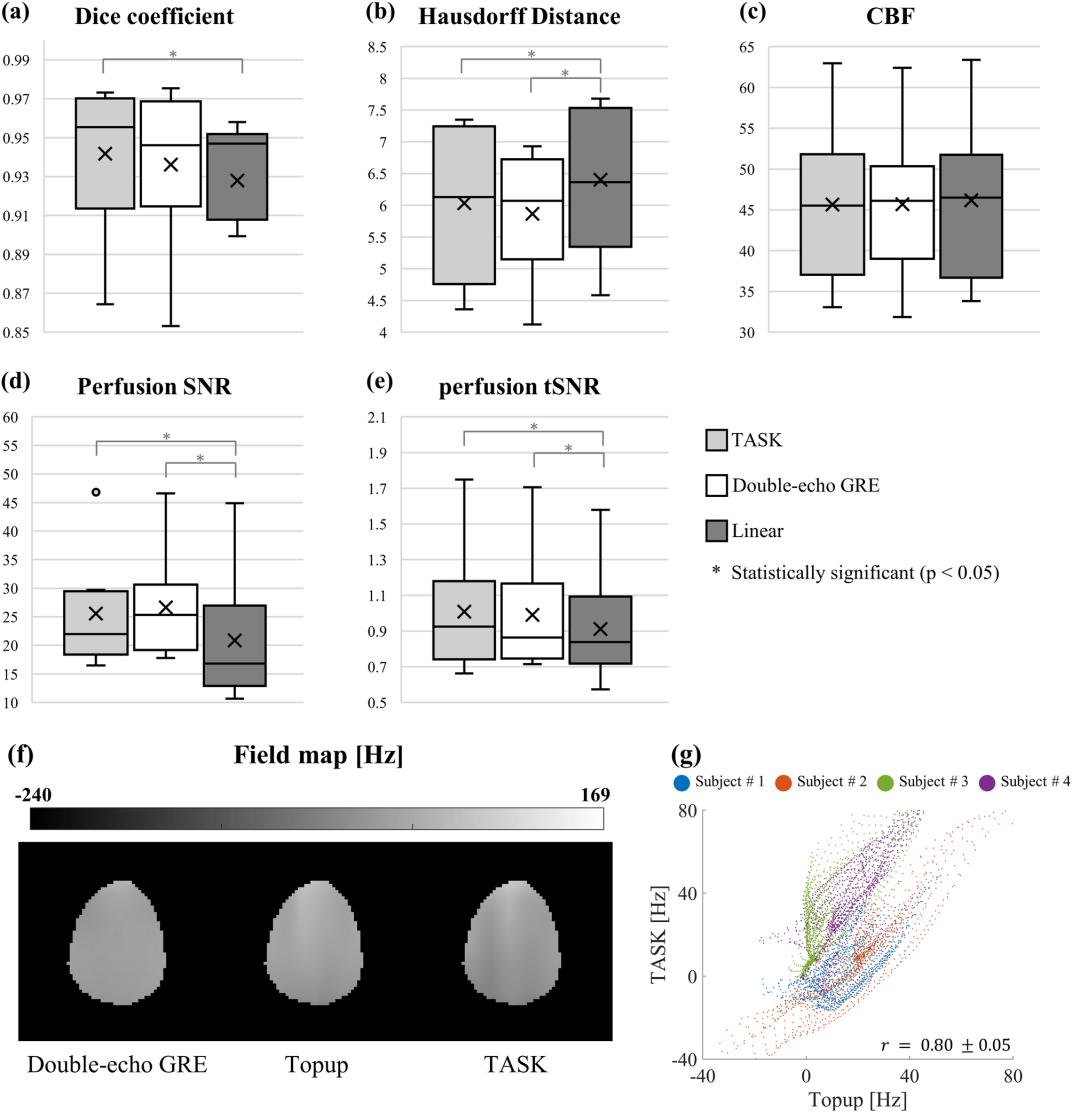
Quantitative comparisons of the corrected images in all subjects and comparisons of the estimated field maps in a representative subject. (**a**) Dice coefficient (five center slices), (**b**) Hausdorff Distance (five center slices), (**c**) CBF (center slice), (**d**) perfusion SNR (center slice) and (**e**) perfusion tSNR (center slice) were measured in the EPI images processed by TASK (TASK, light gray), double-echo GRE method (double-echo GRE, white), and in the linear EPI images (Linear, dark gray). The gray matter mask was used to calculate the average values of the gray matter area. The ground truth brain boundary from GRE image was the reference of the Dice coefficient and Hausdorff distance. For fair comparison, the linear EPI images were not processed through distortion correction under the assumption of no additional B_0_ field mapping. Two-tailed t-test verified the statistical significance. (**f**) The field maps obtained by the TASK, double-echo GRE method, and conventional topup (Topup) of subject #2 are shown. (**g**) The scatter plot of the voxel distribution of the estimated field maps between the center slices of “Topup” and “TASK” from all subjects shows a strong correlation ($r =0.80\pm 0.05$).

For the quantitative comparison, Dice coefficient, Hausdorff distance, CBF, and perfusion SNR and perfusion tSNR were measured for all subjects. (Fig. 9a–e) The Dice coefficient and Hausdorff distance of the TASK were distinguished from that of the linear EPI ($p=0.0028, 0.0115\ll 0.05$), showing the ability of the proposed distortion correction algorithm. Additionally, the Dice coefficient and Hausdorff distance were similar between the TASK and double-echo GRE methods ($p=0.1018, 0.2775\gg 0.05$) indicating good distortion correction performance. The TASK showed CBF values indistinguishable from those of linear EPI ($p=0.579\gg 0.05$), indicating reasonable CBF quantification. Lastly, perfusion SNR and tSNR of the TASK was 22% and 10% higher than that of linear EPI ($p=0.0024, 0.017\ll 0.05$), consistent with the previous research. This shows that the TASK can take advantage of 1sh-CenEPI, which has higher SNR for magnetization prepared imaging because of shorter TE. It should be noted that the TASK and double-echo GRE methods were derived from the same 1sh-CenEPI data, and thus the quantitative values of CBF, perfusion SNR and tSNR were not distinguished between the two methods. Consequently, availability of distortion correction by its own single k-space without additional information was verified quantitatively.

Figure 9f,g shows the qualitative and quantitative correlations among the conventional B_0_ field map estimations and the proposed estimation. The estimated field map (representing information about pixel-wise distortion) by the TASK had a tendency similar to those by the conventional double-echo GRE and the topup methods. Quantitatively, there was strong correlation between the estimated field maps by the TASK and the conventional topup method (Fig. 9g) in the four tested subjects who underwent the conventional topup scan ($r = 0.80 \pm 0.05$).

**Figure 10:**
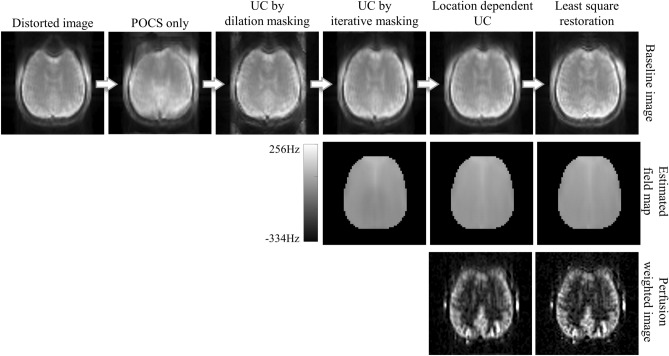
Ablation study of the TASK. The proposed steps were added one by one. POCS only: only POCS was applied with no other steps. UC by dilation masking: uniformity correction with a single sigma value using the mask by dilation from the blip-up mask. UC by iterative masking: iterative procedure of automatic brain masking and uniformity correction with a single sigma value. Location dependent UC: Similar to “UC by iterative masking” but only for the last iteration the sigma values were assigned in a location dependent manner to be 0.5 at the center of brain mass and 8 in the brain boundaries and linearly increasing in between. Least square restoration: “Location dependent UC” with the least-square restoration at the stage of distortion correction.

To show the contributions of each proposed step, the corrected images without each step are presented in Fig. 10. When only POCS was applied without the other steps (i.e., omitting iterative procedure in Fig. 3), the output became more distorted because the signal suppression in the input images disrupted the performance of the topup method. Applying uniformity correction with a single sigma value (i.e., conducting only 1st iteration in Fig. 5) could improve the performance by compensating for the signal suppression to some degree. The distortion correction was improved with the mask by the suggested iterative process (i.e., conducting iterative procedure in Fig. 5 without location dependent sigma) than the mask by dilation from the blip-up mask. However, there were patch-like artifacts on the estimated field map presumably due to remaining signal intensity mismatch between the blip-up and down images, resulting in blurring in the inner structure. Accordingly, the location-dependent uniformity correction was applied for the last iteration (i.e., performing all proposed procedures for field map estimation), which resolved the artifacts in the estimated field map and decreased the blurring in the perfusion-weighted images. Finally, at the stage of obtaining the final corrected images, the least-square-restoration could minimize the blurring further and make the fine structures even clearer. Therefore, Fig. 10 shows the degradation of the results when each of the proposed steps is absent, confirming their contributions.

## Discussion

A new process for EPI distortion correction was presented in this paper. The TASK has advantages over the conventional methods in that it can correct the distortion by its own single k-space information, enabling dynamic B_0_ field mapping. It does not require additional scans such as the B_0_ field mapping, while maintaining temporal resolution and distortion correction performance. Also, any artifacts and problems associated with the time gap between imaging objects and additional scan protocols (e.g., motion artifact, field map drifting) can be resolved. If the B_0_ field map unexpectedly changes, it can be updated with a single k-space scanned after the field map change. Furthermore, it has advantage of not requiring any modification of the existing topup algorithm. It is sufficient to transfer the two extracted inputs by the TASK to the conventional topup algorithm.

The quantitative evaluations confirmed that the TASK worked well in every subject and in different scan parameters. The Dice coefficient and Hausdorff distance quantitatively verified that the correction level was similar to that of the double-echo GRE method. Furthermore, it was shown that the inner structures and CBF values were preserved. Moreover, the qualitative evaluation verified that TASK corrected the distortion to match the ground truth boundary as shown in Figs. 6, 7 and 8. It should be noted that there were differences in contrast between 1sh-CenEPI and the conventional EPI images because of the significantly shorter TE for 1sh-CenEPI, and thus certain structures outside the brain boundary might be more visible in 1sh-CenEPI.

This study considered that the ground truth brain boundary was extracted from the distortion-free GRE image (orange line in Fig. 8) and that the ground truth CBF maps were those from 1sh-CenEPI corrected by the double-echo GRE method. It should be noted that the EPI images for the conventional topup were acquired with linear phase-encoding ordering and longer TE, in contrast to the 1sh-CenEPI which was reconstructed by TASK and the double-echo GRE methods. That is, the EPI images corrected with the conventional topup could not be considered the ground truth. Therefore, the 1sh-CenEPI images corrected using the double-echo GRE method can be regarded as the ground truth, because it was validated in the previous study^12^ and used the same 1sh-CenEPI data as TASK. Nonetheless, it is still difficult to determine superiority of the performance between the TASK and double-echo GRE methods. Therefore, we may need to focus on the similarity of inner structures and CBF quantitative values rather than the differences between the two methods.

Additionally, the B_0_ field maps estimated by the TASK had similar distributions to those of the conventional methods, especially showing strong correlation with those of the conventional topup algorithm. However, the estimated B_0_ field maps by the conventional and proposed topup methods show some band artifacts on the white matter side, compared to the field map estimated by the double-echo GRE method (Fig. 9f). Such artifacts are typically observed when the SNR of inputs is insufficient, often due to factors such as diffusion gradients^33,34^. It is important to emphasize that the aim of this study is to generate the B_0_ field maps using only a single k-space dataset at a level of quality comparable to those generated by the conventional topup algorithm, which typically employs two full k-space datasets with opposite distortions and is widely adopted within the research community.

While there was an overall strong correlation in the estimated B_0_ field maps between TASK and the conventional top-up algorithm, there might be a weaker correlation in specific cases, such as subject #3 as shown in Fig. 9g. This reduced correlation can be understood from three perspectives. Firstly, it’s important to note that the conventional top-up algorithm utilizes inputs from two linear EPI images with opposite distortions, whereas the TASK method relies on a single 1sh-CenEPI image. Consequently, several factors may contribute to lower the correlation between the estimated field maps of TASK and the top-up method. These factors could include B_0_ field drift, subject motion, and differences in scan parameters. Secondly, it’s worth mentioning that the conventional top-up method did not perform effectively in subject #3, as illustrated in Fig. 8a. In other words, the lower correlation with the topup method should not be interpreted as poor performance of the TASK method in general. Lastly, there might be more general factors that lower the correlation in the estimated field maps. The input images for the topup algorithm, derived from linear EPI, and TASK with 1sh-CenEPI, exhibited similar distortion tendencies, albeit with slight differences in the magnitude of distortion (Supplementary Fig. S1). This variation could be attributed to the differences in phase-encoding order; one set of images was acquired with a linear order and a longer echo time (conventional top-up method), while the other was acquired with a centric order (1sh-CenEPI) and a shorter echo time (TASK).

The key idea of this study is extracting two input images for the topup algorithm by separating the single 1sh-CenEPI k-space into two halves. However, it could lead to two problems; (i) signal suppression by incompleteness of partial k-space and (ii) signal mismatch between blip up and down images. The TASK contained the processes to resolve the two problems. The signal mismatch between blip up and down images was resolved by including the center phase-encoding line (not affected by the phase-encoding blip) in both blip up and down k-spaces. Therefore, the TE difference between the two images became nonsignificant, and the signal and contrast differences also became negligible. Furthermore, the uniformity correction helped to overcome the signal suppression. Additionally, the signal distribution between two images became more similar, because the same uniformity correction (the same sigma values) was applied between the two inputs. It should be noted that these processing steps (uniformity correction and inclusion of the center k-space line in both halves) were applied only for the B_0_ field map estimation, and thus would not cause any artificial effect on the final distortion-corrected image. Moreover, it’s worth noting that while the reconstructed input images by TASK may appear to be fully-sampled images, they are, in fact, surrogate images. TASK generates these input images solely for use as inputs in the top-up algorithm.

**Figure 11:**
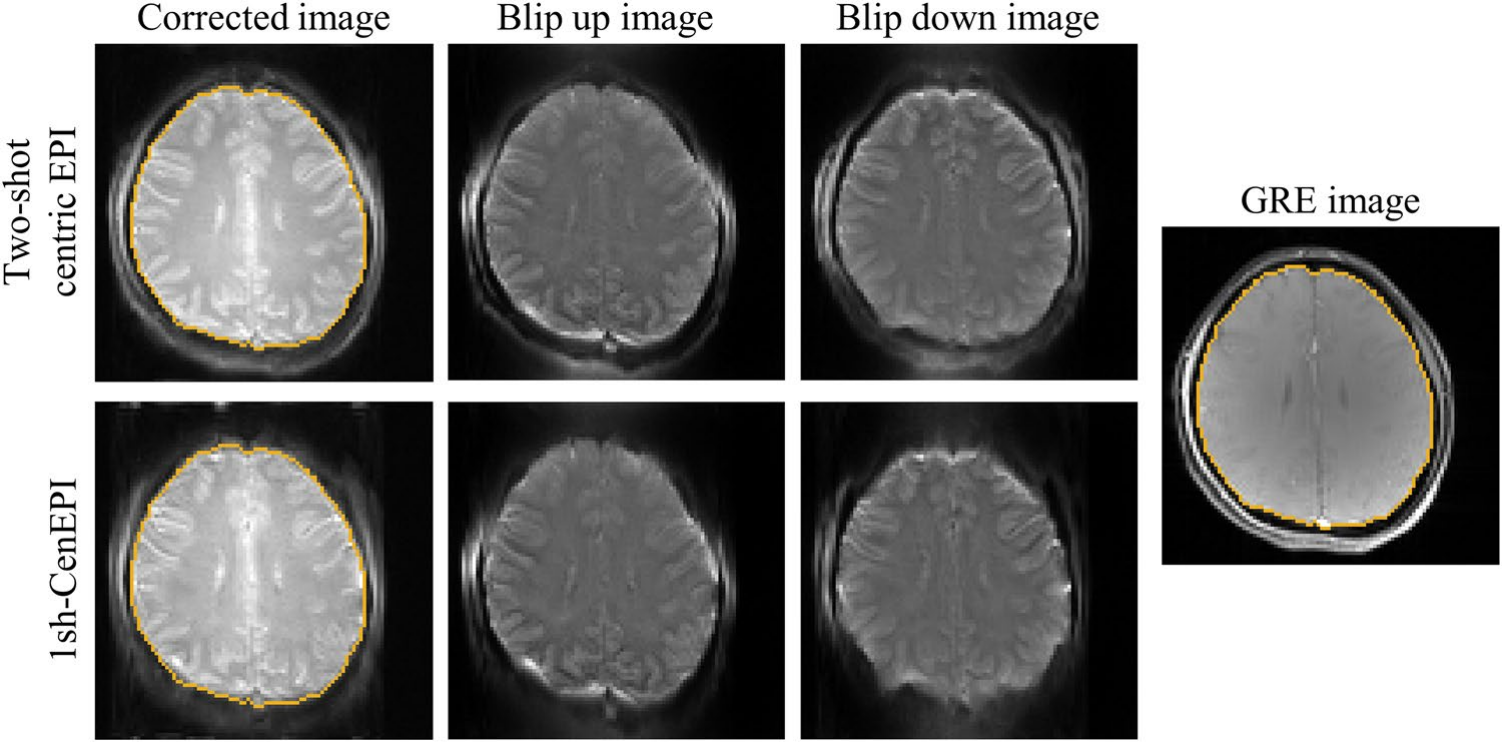
Comparisons of blip up/down images and corrected images by TASK between 1sh-CenEPI and two-shot centric EPI. The blip up and down images show similar distortion tendency and level between 1sh-CenEPI and two-shot centric EPI. It verified that the effect of jump blip on the distortion was compensated and negligible. The slight difference might be caused by eddy current. The comparison experimentally confirmed that 1sh-CenEPI did not have additional distortions caused by jump blips. Furthermore, it verified that the TASK can have wide applicability including being applied to conventional multi-shot centric EPI.

For 1sh-CenEPI to be imaged with centric order in single-shot, a jump blip was applied to every specific number of phase encoding lines (four in this study). Since the jump blips have longer duration and higher gradient magnitudes, it may potentially affect the performance of the proposed distortion correction. To investigate this effect, we compared the blip up and down images of the conventional two-shot centric EPI, which had the same scan parameters as the 1sh-CenEPI but did not have jump blips, with those of 1sh-CenEPI. We observed that the tendency and level of distortion were very similar between two-shot centric EPI and 1sh-CenEPI (Fig. 11). This indicates that the impact of the jump gradients (or the inconsistent bandwidth/echo spacing) on the distortion is negligible. Theoretically, the phase accumulations caused by magnitudes of the jump blips are offset by several blip gradients in the opposite direction, resulting in the same phase accumulation as in conventional EPI. Also, the inconsistent echo-spacing between two adjacent phase-encoding lines in 1sh-CenEPI was resolved through the whole-echo phase correction. This correction compensates for the N/2 ghost by accounting for the inconsistent echo-spacing in the same manner as the main EPI scan, eventually reducing distortion. This was already demonstrated in the previous paper^12^.

Furthermore, the previous paper^12^ demonstrated that Generalized Autocalibrating Partial Parallel Acquisition (GRAPPA) reconstruction worked well in 1sh-CenEPI, indicating that the reconstruction was possible with the same weights in all phase encoding lines, thus suggesting consistent phase accumulation for each line. Moreover, the impact of signal discontinuities between some phase-encoding lines caused by jump blips was previously investigated using phantom and human brain data, as presented in the supplementary material of the previous study^12^. The extent of signal discontinuity, as determined by the number of jump blips, was found to have an insignificant effect on the image distortions. Therefore, it was proved theoretically and experimentally that the contribution of the jump blips on the distortion correction is negligible in the proposed TASK. As a result, the distortion tendency of 1sh-CenEPI is the same as that of conventional EPI, ensuring the same topup performance as the conventional EPI.

The location-dependent uniformity correction along with the automatic brain masking improved the performance of the TASK. The lower sigma values (less smoothing) improved signal homogeneity in the inner brain area (Fig. 4), resulting in mitigation of the patch-like artifact in the estimated field map (Fig. 10). The higher sigma values (more smoothing) reduced the artificial boundary of the brain, complementing imperfect brain masking and thus improving subject robustness (Fig. 4). The location-dependent uniformity correction took both advantages of the high and low sigma values simultaneously; The sigma values were set depending on the relative distance from the center of brain mass and the brain boundaries, making the results consistent across the subjects. Therefore, the TASK was robust to not only imperfect masking but also position and size of the subjects.

Although the TASK was robust to brain masking, it is still important to draw exact brain masks for the blip-up and down images for the location-dependent uniformity correction. Smaller masks would lose the important boundary information, while bigger masks would increase the unexpected signal around the brain boundary. There have been several methods for drawing the masks; Manual drawing is time-consuming and not practical. For the automatic mask drawing, intensity thresholding^23^ and brain extraction tool^24^ have been suggested but they could not consider the signal suppression and distortion in the EPI image from incompleteness of partial Fourier. Therefore, the iterative procedure of brain mask construction was proposed and demonstrated to be effective in this study to draw the relatively exact mask from the preliminarily estimated B_0_ field map.

N4BiasFieldCorrectoin^35^ or other bias field correction methods could be applied instead of the suggested uniformity correction process. These methods also require the brain mask not to spoil the images by drastically increasing the background noise. However, as mentioned above, due to the images from less than half k-space, brain mask could not be generated properly by the existing automatic process. Therefore, the suggested processes, including iterative procedure of masking and location-dependent uniformity correction, were necessary to correct the signal inhomogeneity even in situations where accurate brain masks cannot be obtained.

For wide application, the TASK should be conducted in different imaging parameters. In this study, it was tested mostly on 3D 1sh-CenEPI and additionally on higher resolution 2D 1sh-CenEPI, both of which were demonstrated to be effective (Fig. 7b,c). Furthermore, we expect the TASK would be applicable to more general conditions with different imaging parameters. If the two input images for the topup have the same signal distribution, the topup algorithm would work properly under the specific imaging parameters^3^. In this study, the only factor for the different signal distribution was the different tendency of signal suppression between two input images. The signal suppression appeared in posterior and anterior of the brain in the blip up and down images, respectively. This tendency would be the same across different imaging parameters, because this kind of signal suppression would not be relevant to the imaging parameters but to the location of zero padding for dividing the single k-space into two (i.e., upper or lower)^21^, which was supported by the data in this study (3D EPI and higher resolution 2D EPI) (data not shown). The location-dependent uniformity correction was empirically regulated to compensate for this common signal suppression tendency. Therefore, the TASK would not require any further optimization process that depends on imaging conditions, subjects and parameters.

Moreover, the TASK can be applied to conventional multi-shot EPI imaging where each half of the k-space has an opposite blip direction, such as two-shot centric EPI^13^. In fact, two-shot centric EPI image with 96 base resolution was corrected well by the proposed distortion correction process (Fig. 11). It shows that the TASK is not limited to 1sh-CenEPI. Additionally, since it can be applied to two-shot centric EPI images that have already been scanned, it offers the advantage of expanding the application to existing data. Furthermore, parallel imaging techniques or simultaneous multi-slice acquisition can be applied as exemplified with GRAPPA in the previous study^12^, leading to possibility of reducing the readout time or obtaining higher resolution images.

In the ablation study, it was confirmed that implementation of every proposed step provided the best outcome. The iterative brain masking could produce a more accurate mask necessary to overcome the signal suppression. The location-dependent uniformity correction contributed to the subject robustness and surmounting the possibility of imprecise brain masking, while improving the quality of the estimated B_0_ field map. Lastly, the least-square restoration^27^ could eliminate the blurring issue. Consequently, all the proposed steps play an important role in the distortion correction.

Additionally, the TASK has a secondary advantage from the benefit of 1sh-CenEPI^12^, where the EPI k-space is filled from the center to the periphery by a single RF excitation. Due to the single-shot centric imaging, it can save scan time in magnetization-prepared imaging compared to other multi-shot centric ordering, which requires multiple magnetization preparations. Therefore, it has at least twice higher temporal resolution compared to other magnetization-prepared EPI with centric order^12^. In addition, since 1sh-CenEPI scans the center part of the k-space first, perfusion SNR and tSNR could be increased compared to the linear-ordered EPI (Fig. 9d,e), despite the smaller TE difference between the linear and 1sh-CenEPI in this study (compared to the previous study^12^) due to partial Fourier applied to the linear-ordered EPI. As base resolution increases and TE gets longer, the SNR difference between linear and 1sh-CenEPI will become larger even with partial Fourier.

In this study, we estimated the B_0_ field map from the first-measured MT-free 1sh-CenEPI, which was used to correct distortions in all the related 1sh-CenEPI images (with and without the pCASL scheme). Dynamic B_0_ field mapping was not applied to the perfusion-weighted images (i.e., 1sh-CenEPI images with the pCASL scheme) because the background suppression decreased the signal intensity, which could potentially lead to poor topup performance. Implementing dynamic B_0_ field mapping for distortion correction requires modification to the existing FSL module for practical utility as well. In a separate experiment, we tested dynamic field mapping with the existing FSL module through multiple tedious manual applications and combinations, which yielded results similar to or slightly better than the B_0_ mapping from the first measurement in some datasets (Supplementary Fig. S2). However, the systematic implementation and evaluation of dynamic B_0_ field mapping with practical utility are beyond the scope of the current study.

The proposed TASK is a promising distortion correction method with efficiency in SNR and temporal resolution, but there are still some limitations to be resolved. First, TASK can only be applied when upper and lower k-spaces have opposite blip directions, such as 1sh-CenEPI or centric EPI. However, as long as this condition is satisfied, it also has the potentials to be applicable even when centric ordering is not used. Secondly, since TASK is based on an EPI image acquired with opposite phase-encoding blips (resulting in opposite distortions), blurring may occur in the resulting image under conditions of inaccurate distortion correction. Lastly, TASK involves relatively complex processes. The processes may be simplified further for better applicability in the future.

## Conclusion

In this study, we proposed a method called “TASK” to correct the EPI distortion by its own single k-space information only. This method corrects the distortion by the unique feature of 1sh-CenEPI without additional measurement of B_0_ field map while maintaining temporal resolution. Furthermore, it can take advantage of 1sh-CenEPI^12^ that has higher SNR. Therefore, the TASK can be a good distortion correction algorithm for magnetization-prepared imaging such as arterial spin labeling^16^, diffusion^36^ and chemical exchange saturation transfer^37^. Since the perfusion maps were corrected well without losing inner detailed structures and having higher SNR in this study, the TASK would be efficient to other magnetization-prepared imaging. Furthermore, the TASK can be widely applied, including conventional two-shot centric EPI.

### Supplementary Information

Supplementary Figures.

## Data Availability

The relevant data can be available from the corresponding author on the reasonable request.
