# Supplementary material for: Distortion correction using topup algorithm by single k-space (TASK) for echo planar imaging
Source: Sci Rep. 2023 Oct 31;13:18751. doi: 10.1038/s41598-023-46163-3 (PMC10618273; doi:10.1038/s41598-023-46163-3)
Supplement: Supplementary file 1 — Supplementary Figures. [file 41598_2023_46163_MOESM1_ESM.pdf]

Distortion correction using topup algorithm by single k-space (TASK) for echo planar imaging

Supplementary Information

Seon-Ha Hwang, Hyun-Soo Lee, Seung Hong Choi, Sung-Hong Park*

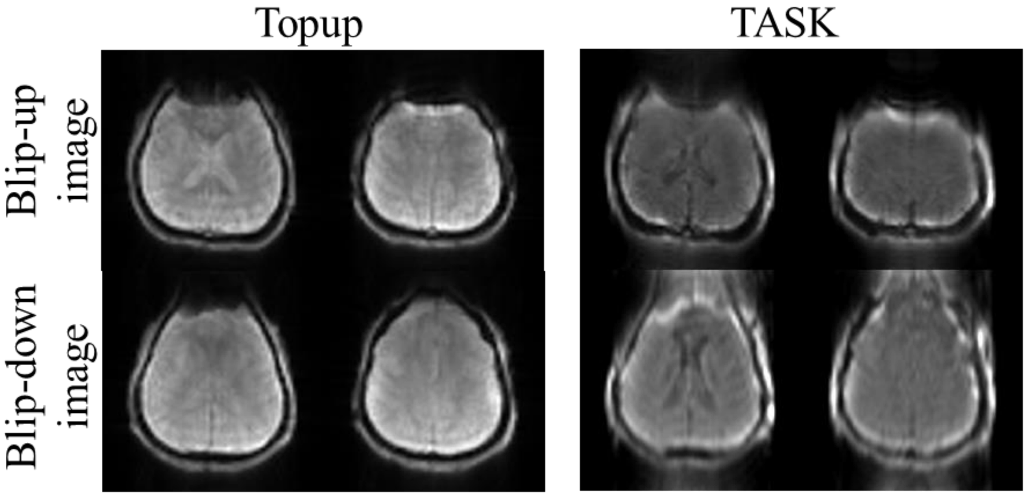

Supplementary Figure S1 Comparison of input images of subject #3 between conventional topup (Topup) and TASK.

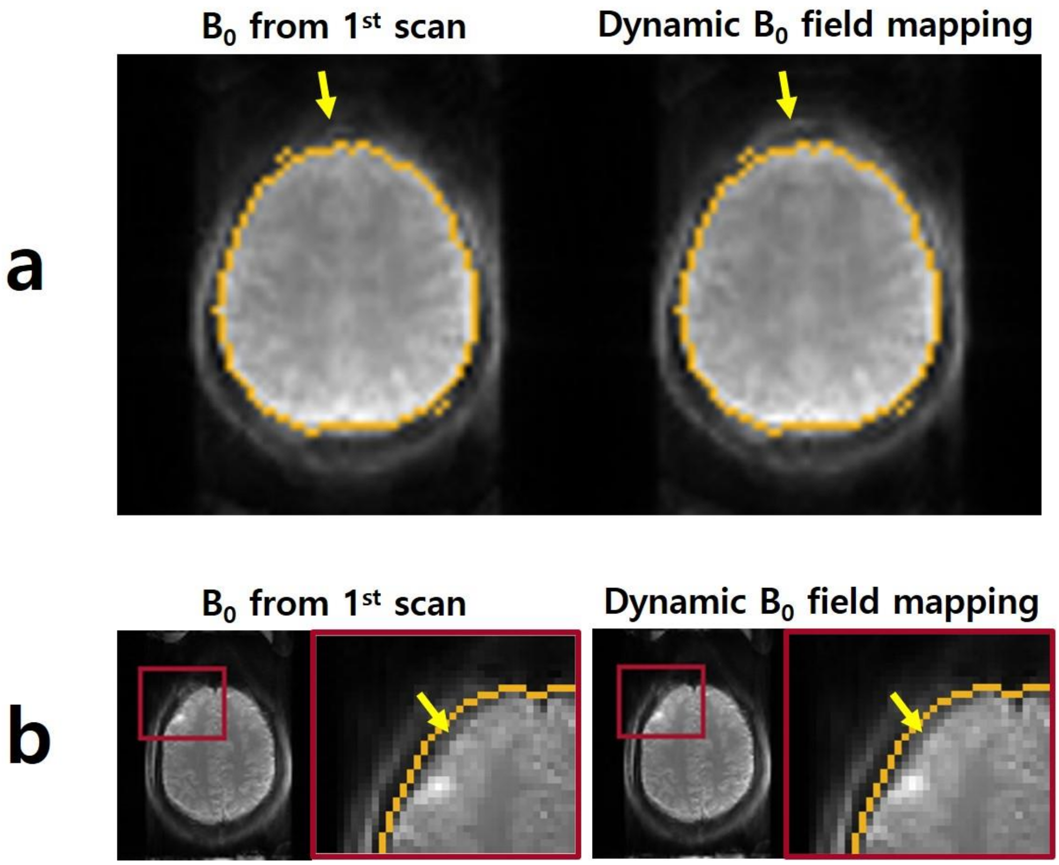

Supplementary Figure S2 Comparison of distortion correction of the 1sh-CenEPI images with the B_0 field from the first measurement and with dynamic B_0 field mapping. The results from 3D 1sh-CenEPI (**a**) and 2D 1sh-CenEPI (**b**) are shown. The arrows indicate the regions with slightly better correction performance.
